# Size effect on atomic structure in low-dimensional Cu-Zr amorphous systems

**DOI:** 10.1038/s41598-017-07708-5

**Published:** 2017-08-04

**Authors:** W. B. Zhang, J. Liu, S. H. Lu, H. Zhang, H. Wang, X. D. Wang, Q. P. Cao, D. X. Zhang, J. Z. Jiang

**Affiliations:** 1 International Center for New-Structured Materials (ICNSM), Laboratory of New-Structured Materials, State Key Laboratory of Silicon Materials, and School of Materials Science and Engineering, Zhejiang University, Hangzhou, 310027 People’s Republic of China; 2 School of Materials Science and Engineering, Zhejiang University of Technology, Hangzhou, 310014 People’s Republic of China; 3 Department of Chemical and Materials Engineering, University of Alberta, Edmonton, Alberta T6G 2V4 Canada; 4 Institute of Nanosurface Science and Engineering, Shenzhen University, Shenzhen, 518060 People’s Republic of China; 5 State Key Laboratory of Modern Optical Instrumentation, Zhejiang University, Hangzhou, 310027 People’s Republic of China

## Abstract

The size effect on atomic structure of a Cu_64_Zr_36_ amorphous system, including zero-dimensional small-size amorphous particles (SSAPs) and two-dimensional small-size amorphous films (SSAFs) together with bulk sample was investigated by molecular dynamics simulations. We revealed that sample size strongly affects local atomic structure in both Cu_64_Zr_36_ SSAPs and SSAFs, which are composed of core and shell (surface) components. Compared with core component, the shell component of SSAPs has lower average coordination number and average bond length, higher degree of ordering, and lower packing density due to the segregation of Cu atoms on the shell of Cu_64_Zr_36_ SSAPs. These atomic structure differences in SSAPs with various sizes result in different glass transition temperatures, in which the glass transition temperature for the shell component is found to be 577 K, which is much lower than 910 K for the core component. We further extended the size effect on the structure and glasses transition temperature to Cu_64_Zr_36_ SSAFs, and revealed that the *T*_g_ decreases when SSAFs becomes thinner due to the following factors: different dynamic motion (mean square displacement), different density of core and surface and Cu segregation on the surface of SSAFs. The obtained results here are different from the results for the size effect on atomic structure of nanometer-sized crystalline metallic alloys.

## Introduction

The atomic structure of metallic glasses (MGs) is a long-standing unsolved issue in condensed matter community^1–3^. Considerable efforts have been made to describe the structure and/or to reveal various factors influencing atomic structure of metallic glasses by experiments^4, 5^ and simulations^6, 7^, such as cooling rate^8–10^ and composition^11–13^. The sample size effect on properties of MGs has been reported, e.g., size effect on stability^14^, strength^15–18^, ductility^19–21^, elastic limit^22–24^, plasticity and deformation mode^25–30^. However, the size effect on atomic structure of low-dimensional amorphous systems, including zero-dimensional small-size amorphous particles (SSAPs) and two-dimensional small-size amorphous thin films (SSAFs) has not been systematically reported yet, possibly because the precise fabrication of free standing SSAPs and SSAFs with various sizes, while keeping the thermal history invariant, is rather difficult if not impossible. In crystalline metallic particles, the smaller the particles, the stronger the sample size effect on atomic structure. Atomistic simulations have been widely applied to study the structure of nanometer-sized crystalline metallic particles^31–35^ and thin films^36, 37^. Thus, we resort to atomistic simulations to study the size-dependent atomic structure in SSAPs and SSAFs. In this work, we report the results of atomic structures in a zero-dimensional Cu_64_Zr_36_ SSAPs, ranging from about 11 Å to 60 Å in diameter, and in a two-dimensional Cu_64_Zr_36_ SSAFs, ranging from 8.6 Å to 61.5 Å in thickness as a prototype model system in which the Cu-Zr potential was well developed and applied^27–30, 38–41^, by molecular dynamics simulations. It is found that sample size strongly affects local atomic structure in Cu_64_Zr_36_ SSAPs and SSAFs, which are composed of core and shell components in SSAPs and of core and surface layers in SSAFs. Each component has different atomic packing structure, average coordination number, bond length, and the degree of ordering. These atomic structure differences in Cu_64_Zr_36_ SSAPs and SSAFs with various sizes or thicknesses result in different glass transition temperatures, in which the glass transition temperature for the shell component is found to be 577 K, which is much lower than 910 K for the core component in SSAPs while *T*_g_ decreases with the thickness of SSAFs. It has been manifested unambiguously that free surface is the dominant cause for *T*_g_ reductions. Cu atoms segregate to the surface causing a far higher concentration while the core component almost remains the same composition as films. The finding obtained here will trigger more studies on the unsolved puzzle of atomic structure in disordered materials in general.

## Results and Discussion

### Pair distribution functions in Cu_64_Zr_36_ SSAPs

**Figure 1:**
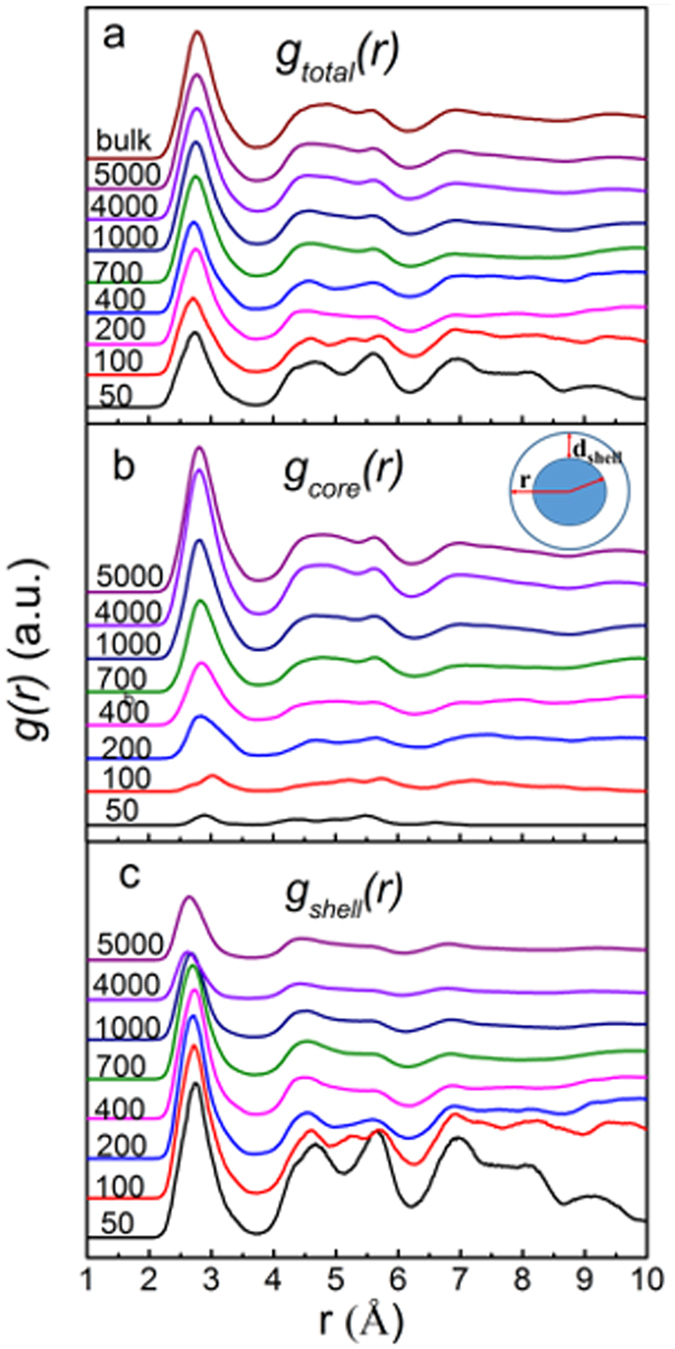
Pair distribution functions *g*(*r*) for Cu_64_Zr_36_ SSAPs with various sizes together with bulk sample for the (**a**) total, (**b**) core component together with an illustration of the core-and-shell model in the inset and (**c**) shell component.

**Figure 2:**
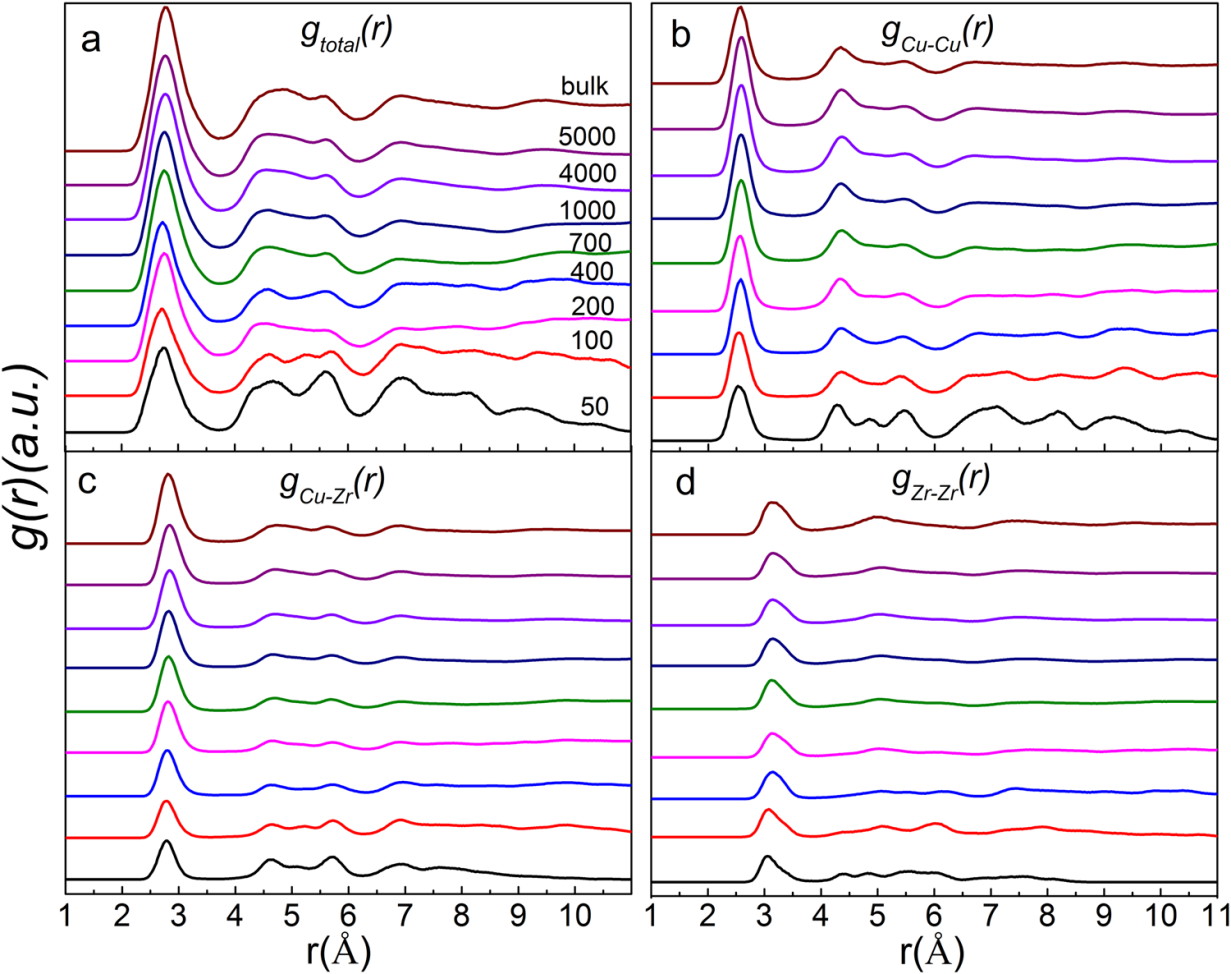
Pair distribution functions *g*(*r*) for Cu_64_Zr_36_ SSAPs with various sizes together with bulk sample for the (**a**) total, (**b**) Cu-Cu partial, (**c**) Cu-Zr partial, (**d**) Zr-Zr partial.

For Cu_64_Zr_36_ SSAPs with various sizes together with bulk sample at *T* = 300 K were plotted in Fig. 1a while partial pair distribution functions for Cu_64_Zr_36_ SSAPs with various sizes are plotted in Fig. 2. The *g*(*r*) of Cu_64_Zr_36_ SSAPs even for a size of 50 atoms still exhibit a relative sharp first peak and a split second peak, which were characteristic of amorphous. The intensity of the first peak increases with the size, while the *g*(*r*) for larger sized Cu_64_Zr_36_ SSAPs (4000 and 5000 atoms) becomes similar to that of bulk Cu_64_Zr_36_ MG. In the range of 4–6 Å, i.e., for the second and third neighbors, *g*(*r*) are different from 50 atoms to 700 atoms, while after 700 atoms, they are similar to the bulk sample. The partial pair distribution functions *g*(*r*) of Cu-Cu, Cu-Zr, and Zr-Zr pairs in Fig. 2 show the increase in intensity of the first peak with sample size while the positions of the peaks in partial *g*(*r*) remain almost unchanged, indicating the average atomic bonds are not sensitive to particle size. The first peak distance becomes larger as particle size increases. A similar observation is also found in nanometer-sized crystalline ZnS particles^42, 43^. To dig out details of local atomic structure in particles, the core-and-shell model in the inset of Fig. 1b is applied, in which the thickness of shell was estimated by the minimum after the first peak in total *g*(*r*) to be about 3.7 Å. In general, it is tough to select the shell layer thickness for nanometer-sized particles, which depends on materials and properties one selected. From the atomic packing short-range order point of view, using the first minimum in the *g*(*r*) here is not an unreasonable value^14^. It should be mentioned that normally the thickness of the shell could also be a function of particle size. However, the minimum position after the first peak in total *g*(*r*) in Fig. 1a is roughly unchanged by varying particle sizes. Corresponding pair distribution functions *g*(*r*) for the core and shell components are plotted in Fig. 1b and c, respectively. For the 50-atom particle, almost all atoms are located in the shell component, reflected by the similar *g*(*r*) for both total and shell. By increasing particle size, the fraction of atoms in the core component increases. From 50 to 700 atoms, the *g*(*r*) in the range of 4–6 Å largely change in both core and shell components. Above 700 atoms, *g*(*r*) for the core component are similar to the total *g*(*r*), while *g*(*r*) for the shell component does not change much for 1000, 4000 and 5000 atoms. It is found that the first peak positions in *g*(*r*) for the core component are larger than those for the shell component in Table 1, revealing larger average bond lengths in the core component than those in the shell component, which might be linked with different coordination numbers and/or compositions in shell and core components, discussed later. It shows that the total average bond length has a tend to decrease with the size decreasing of nanoparticles from 2.78 Å to 2.73 Å, which means that the nanoparticle shrunk. It can be concluded from pair distribution functions that atomic structures in both core and shell components largely differ in Cu_64_Zr_36_ SSAPs with sizes from 50 to 700 atoms while atomic structures in particles with sizes above 1000 atoms are similar.

**Table 1:** The position and average coordination number of the first peak in g(r) for Cu_64_Zr_36_ SSAPs total sample, and core and shell components.

Components Parameters		Number of atoms							
		50	100	200	400	700	1000	4000	5000
Total	Position (Å) (±0.01)	2.74	2.72	2.73	2.75	2.76	2.76	2.78	2.78
	CN (±0.1)	8.2	9.1	9.9	10.4	10.8	11.0	11.7	11.8
core	Position (Å) (±0.01)	2.89	3.01	2.90	2.87	2.86	2.83	2.82	2.82
	CN (±0.1)	11.8	12.8	13.1	12.9	12.8	12.6	12.6	12.3
shell	Position (Å) (±0.01)	2.74	2.71	2.70	2.71	2.70	2.68	2.62	2.66
	CN (±0.1)	8.1	8.7	8.9	9.2	9.1	8.6	8.5	8.4

### Coordination number distributions and short range atomic packing in Cu_64_Zr_36_ SSAPs

**Figure 3:**
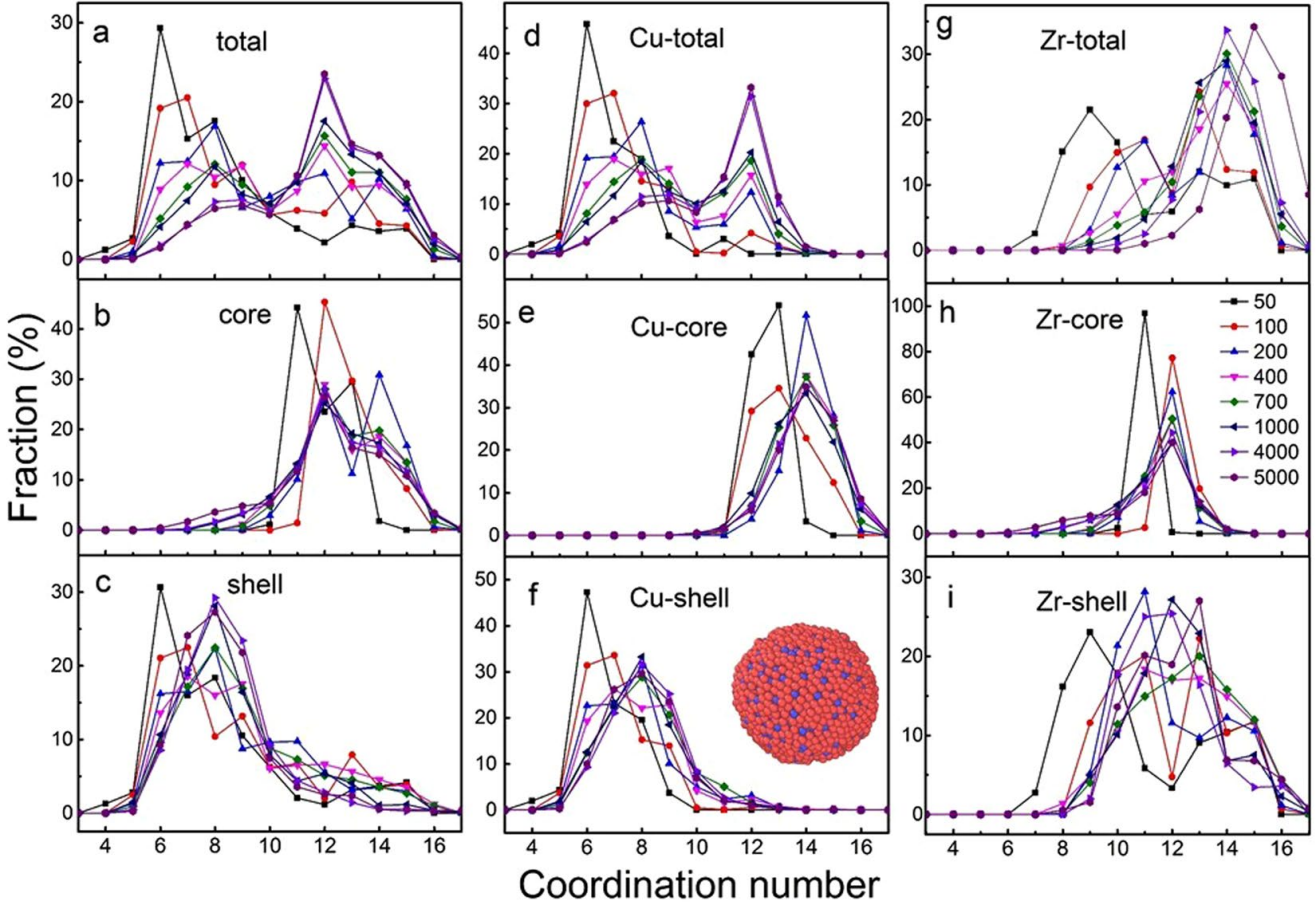
The coordination number distributions of the total sample, core and shell components, (**a–c**) for both elements, (**d–f**) for only Cu-centered atoms, and (**g–i**) for only Zr-centered atoms, respectively. The inset in (**f**) is the 5000-atom particle with red balls as Cu atoms and blue balls as Zr atoms.

**Figure 4:**
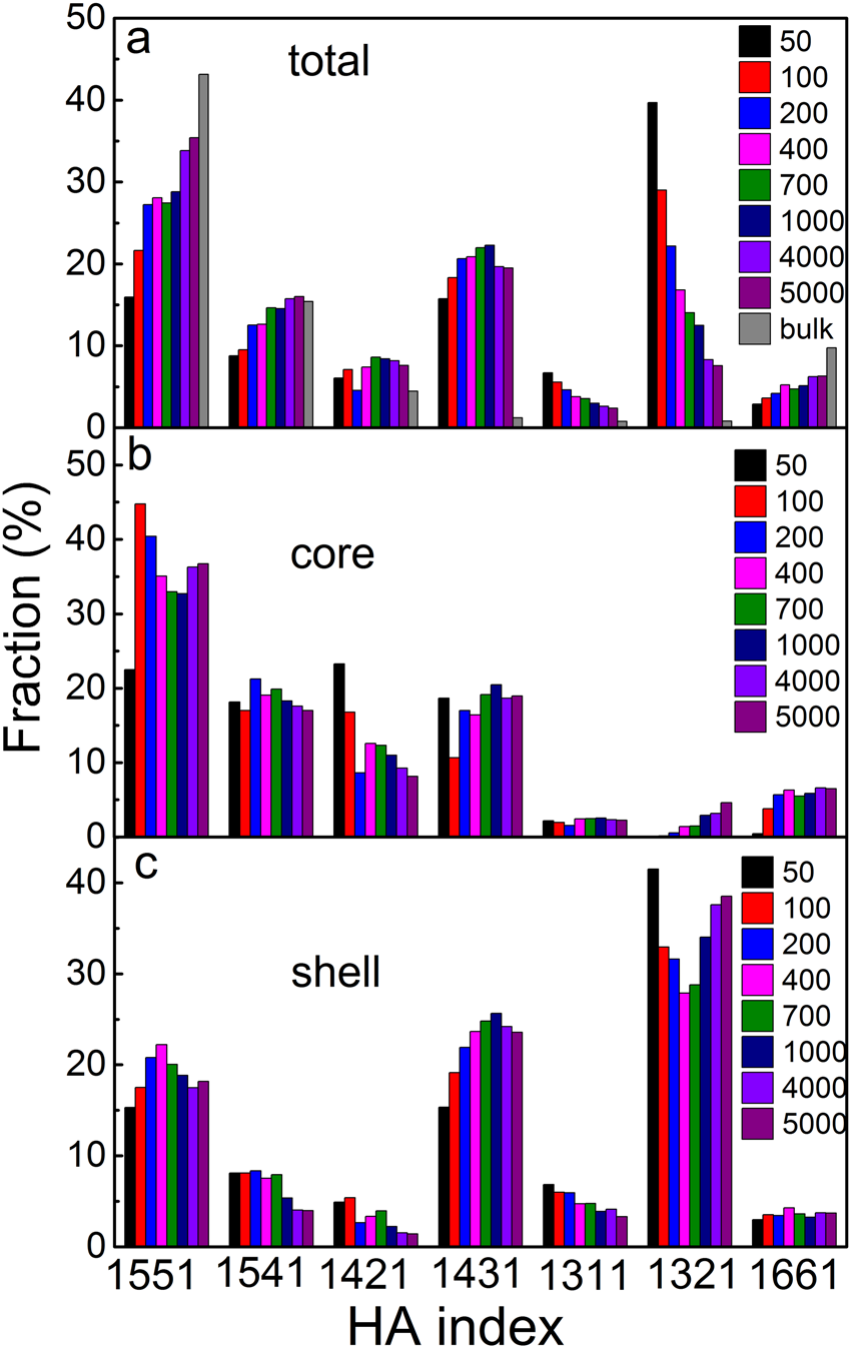
The HA index of Cu_64_Zr_36_ SSAPs together with bulk sample. (**a**) Total sample, (**b**) core component, and (**c**) shell component.

The coordination number (CN) distribution of the total particle, and core and shell components in Cu_64_Zr_36_ SSAPs are further calculated in Fig. 3. The CN distribution in total particles show a bi-peak distribution centered at about CN = 7 and 12, which strongly depends on the size of SSAPs. With increasing the particle size, the fraction of atoms with high CN increase at the expense of the fraction of atoms with low CN. The bi-peak distributions are clearly linked with the CN distributions of the core and shell components in Fig. 3b and c, respectively. In the core component, CN distributes in the range of CN = 10–16 while in the shell component, it distributes from CN = 5 to CN = 10. This lower CN in the shell component can be explained by the surface effect while higher CN in the core component reveals relative dense atomic packing in Cu_64_Zr_36_ SSAPs. Similar to *g*(*r*), it is found that CN distribution in both core and shell components strongly depend on the particle size in the range 50–700 atoms while it remains almost steady for particle size larger than 700 atoms. In terms of Cu- and Zr-centered surroundings in total particles, core and shell components in Fig. 4d–i, the CN of Cu atoms in the core component is centered at about 12 and about 14 for Zr atoms. However, the CN of Zr atoms in the shell component still have higher CN (>9) compared with Cu atoms (CN < 9), which is caused by the fact that the outmost surface is found to be mainly Cu atoms in the inset in Fig. 3f, i.e., Zr atoms are located at the inner parts of the shell defined here. This suggests that Cu atoms could be segregated into the shell of Cu_64_Zr_36_ SSAPs^14^, resulting in smaller average bond length in the shell component because of smaller Cu-Cu bond length as compared to Cu-Zr and Zr-Zr bonds. Similar Cu segregation at the top surface layer is also observed in Cu_64_Zr_36_ SSAFs. Similar works for the segregation effect were recently reported^44, 45^.

To further understand atomic packing in Cu_64_Zr_36_ SSAPs, Honeycutt-Anderson (HA) index is introduced to characterize the common neighbors of an atom pair^46^, which has four indices *i, j, l, m*. If atom A and atom B form a bond, *i* = 1, and otherwise *i* = 2. *j* denotes the number of common neighbors which form bonds with atom A and atom B. *l* represents the number of bonds formed among the neighboring atoms. *m* is a special index to classifying the bonds arrangements. Figure 4 shows the HA index of total particle, core and shell components. In Cu_64_Zr_36_ SSAPs, the 1551 HA index, which is characteristic of the icosahedral cluster, together with the 1541 and 1431 HA index, which are assigned to be icosahedral-like structure, have considerable percentages in both total particle and the core component, implying that icosahedral with fivefold symmetry are dominating in Cu_64_Zr_36_ SSAPs, similar with nanometer-sized metallic crystalline particles^31, 35^. The dominant HA index in the shell component is 1321, which is rarely found in the core component with a possible reason that atoms in shell do not have enough atoms to form bonds and share common neighbors, followed by 1431 and 1551 HA index in the shell component. It should be mentioned that on the surface of nanometer-sized metallic crystalline particles^31, 35, 47^, icosahedral atomic packing was still dominant, which is different from the results obtained here for nanometer-sized metallic glassy particles. In the total particle, with increasing the particle size, the fractions of 1551 and 1541 index increase, close to bulk Cu_64_Zr_36_ MG, while 1321 index decreases, revealing different atomic packing in both core and shell components.

Bond-orientation order parameters, e.g., *Q*_*6*_ and *Q*_*4*_, which are often used to measure atomic cluster symmetries in disordered systems^48–51^. The spherical harmonics analysis was firstly introduced by Steinhardt *et al*.^48^ and the coarse-grained form was adapted by Lechner^52^. The (2 *l* + 1) dimensional complex vector $({q}_{l})$ can be defined for each atom *i* as presented in the Eq. (1).

1 $${q}_{lm}(i)=\frac{1}{{N}_{b}(i)}\sum _{j=1}^{{N}_{b}(i)}{Y}_{lm}({\mathop{r}\limits^{\wedge }}_{ij})$$

where *l* is the free integer parameter, *m* is an integer that runs from −*l* to *l*, ${Y}_{lm}$ are the spherical harmonics, ${\mathop{r}\limits^{\wedge }}_{ij}$ is the vector from atom *i* to atom *j*, and the sum goes over all neighboring atoms ${N}_{b}(i)$ of atom *i*. We could average the spatially local bond order parameters according to the equation ${\mathop{q}\limits^{\wedge }}_{lm}(i)=\frac{1}{{N}_{b}(i)}{\sum }_{j=1}^{{N}_{b}(i)}{q}_{lm}(k)$, and construct the rotationally invariant quantities, as demonstrated in Eq.

2 $${Q}_{l}(i)=\sqrt{4\pi /(2l+1)}|{\mathop{q}\limits^{\wedge }}_{lm}(i)|$$

**Figure 5:**
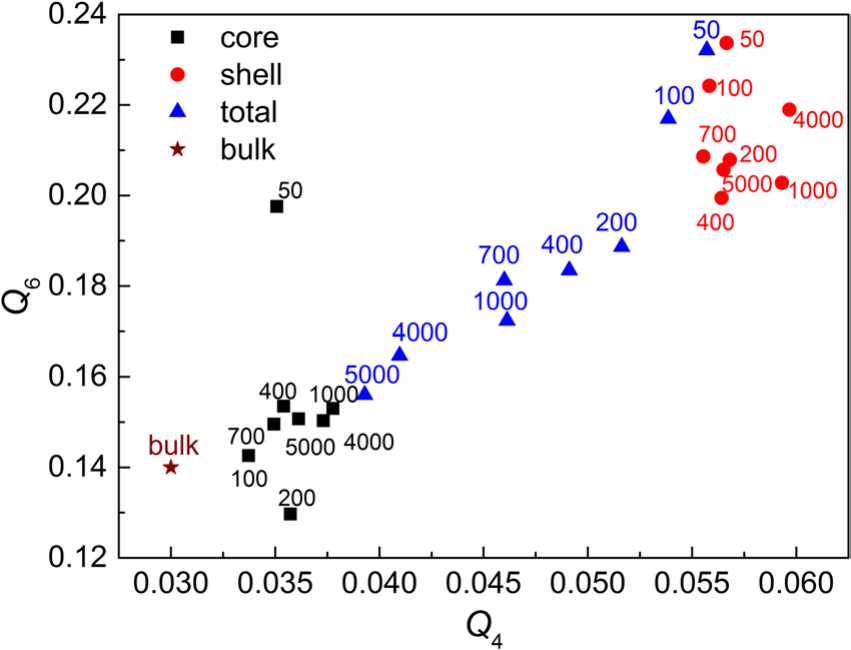
The *Q*_*6*_-*Q*_*4*_ distribution of Cu_64_Zr_36_ SSAPs (total, core and shell components) with various sizes together with the bulk sample. Numbers of atoms are marked.

It should be noted that Mickel *et al*., pointed out the two flaws of Steinhardt order parameters: one is the definition of the nearest neighboring spheres around a given central particle, the other is the discontinuous function of the particle coordinates^53^. After the modification by Lechner, such two shortcomings are avoided by averaging the first neighbor shell of a given particle and the particle itself. The averaged parameters (BOO) are very sensitive to measure the different bond-orientational symmetries and differentiate the local atomic environment. Figure 5 shows *Q*_*6*_ and *Q*_*4*_ values obtained for Cu_64_Zr_36_ SSAPs with various sizes. It is clear that cluster symmetries of the core component largely differ from those in the shell component. Relative higher *Q*_*6*_ and *Q*_*4*_ values, closer to the crystal-like symmetries^54^, are found in the shell component while they are smaller in the core component, close to bulk sample. With increasing particle size, *Q*_*6*_ and *Q*_*4*_ values in total particle gradually decrease with size, most likely due to the gradual increase of the fraction of the core component in Cu_64_Zr_36_ SSAPs.

### Size-dependent glass transition temperature in Cu_64_Zr_36_ SSAPs

**Figure 6:**
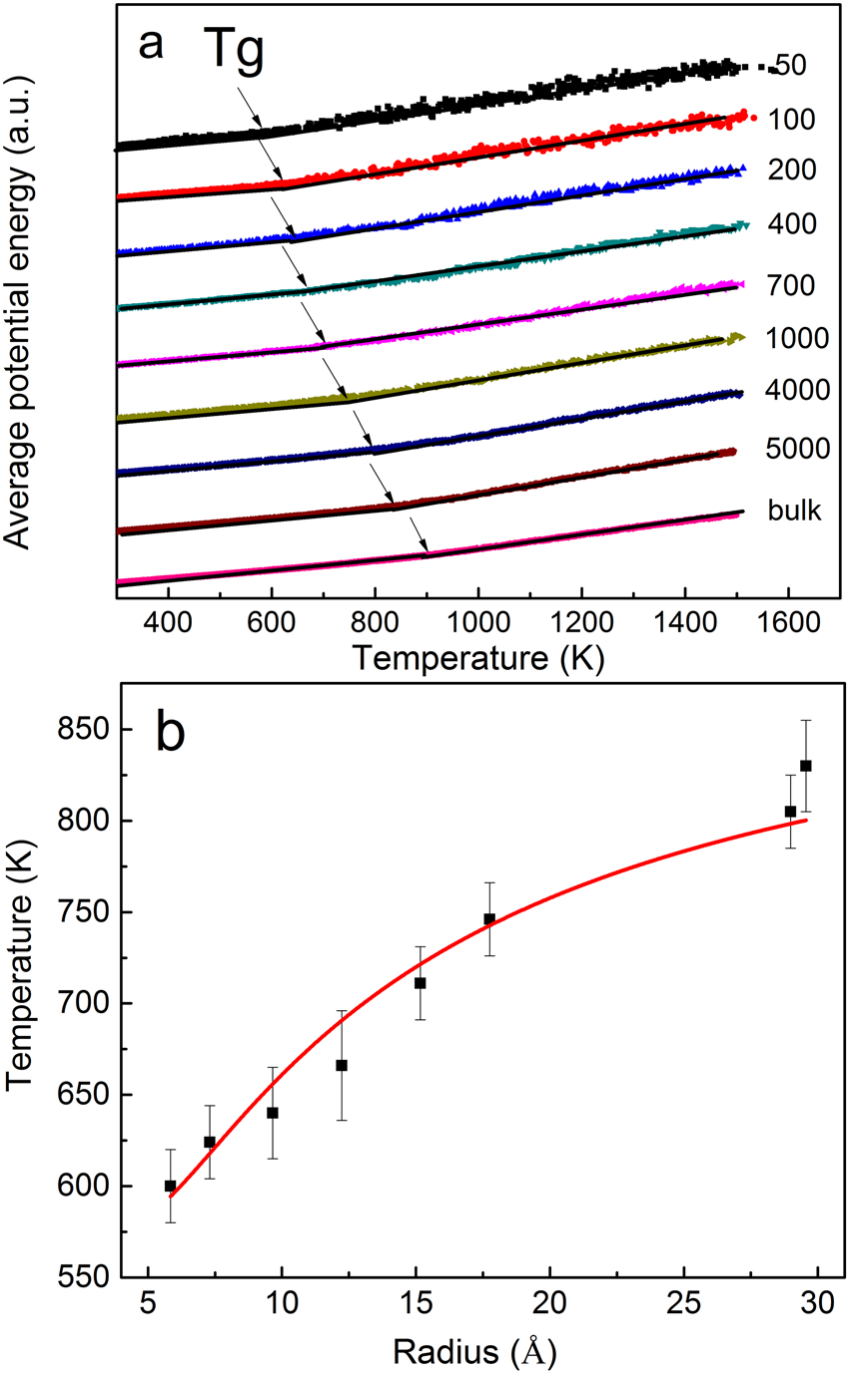
(**a**) Determination of glass transition temperature *T*_g_ via an intersection of low- and high-temperature dependences of the potential energy in Cu_64_Zr_36_ SSAPs with different sizes and bulk Cu_64_Zr_36_ MG. (**b**) The sample-size dependent *T*_g_ of Cu_64_Zr_36_ SSAPs with different sizes together with the fitting curve, which is explained in text.

Above-mentioned results clearly reveal that particle size can largely affect atomic structure of SSAPs. From the structure-property correlation, thus, one expects to have a size effect on properties of such SSAPs. Here, we further studied the size dependence of glass transition temperature, *T*_g_, which is an important problem closely related with atomic structure in disordered systems^52^. The glass transition temperature of SSAPs with various sizes and together with bulk sample, estimated by an intersection of low- and high-temperature dependences of the system potential energy was plotted in Fig. 6a. *T*_g_ increases from 600 K for the 50-atom particle to 825 K for the 5000-atom particle which is close to the value of 910 K for the bulk sample. The sample size indeed influences the glass transition temperature of SSAPs. These *T*_g_ data of all studied SSAPs can be well fitted with a simple equation of *f*_s_ and *f*_*c*_ represent the fractions of shell and core components, and *T*_*g*_^*s*^ and *T*_g_^c^ are glass transition temperatures for shell and core components, respectively. The solid line as plotted in Fig. 6b is the fitting curve with a fitting parameter of *T*_*g*_^*s*^ by assuming *T*_g_^c^ equals to the value for bulk Cu_64_Zr_36_ MG. *T*_*g*_^*s*^ is found to be 577 K, much lower than the 910 K for the core component, which reveals the surface influence to the thermal properties, i.e., the higher the surface-to-volume ratio, the lower the glass transition temperature of studied SSAPs. In addition, the good agreement using fixed glass transition temperatures for both shell and core components indicates that dynamic behaviors of the both shell and core components could be approximately size independence although the *g*(*r*) are size dependence in SSAPs below 700 atoms in Fig. 1.

## Cu_64_Zr_36_ SSAFs

### Pair distribution functions and size-dependent glass transition temperature in Cu_64_Zr_36_ SSAFs

**Figure 7:**
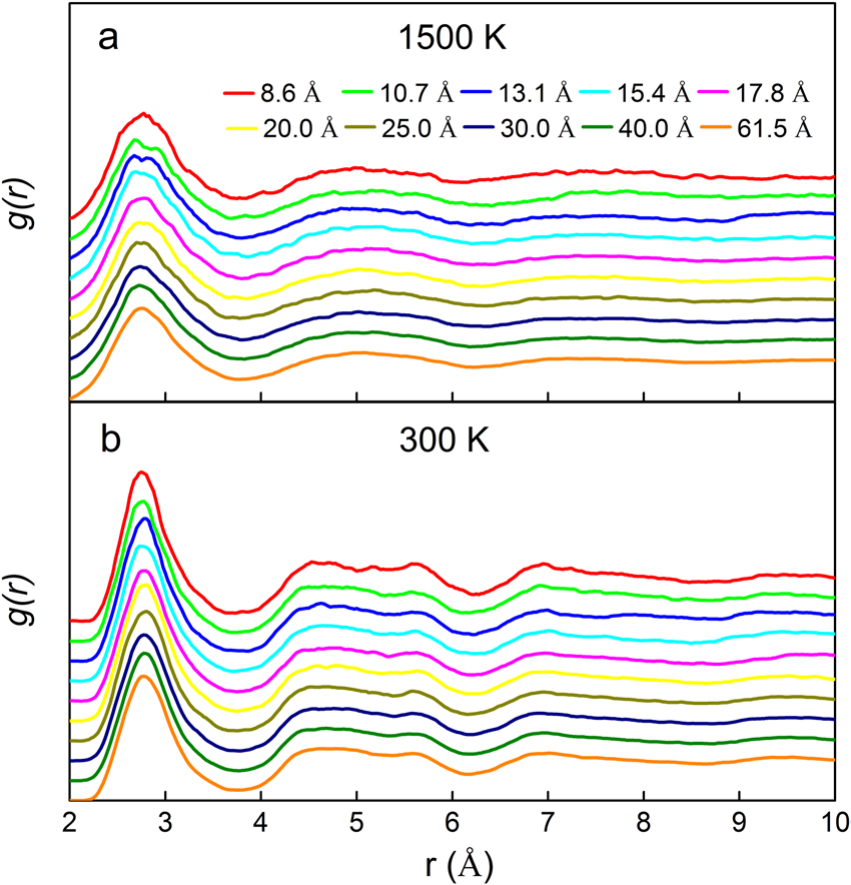
Pair distribution functions *g(r)* for Cu_64_Zr_36_ SSAFs with various thicknesses at (**a**) 1500 K and (**b**) 300 K.

**Figure 8:**
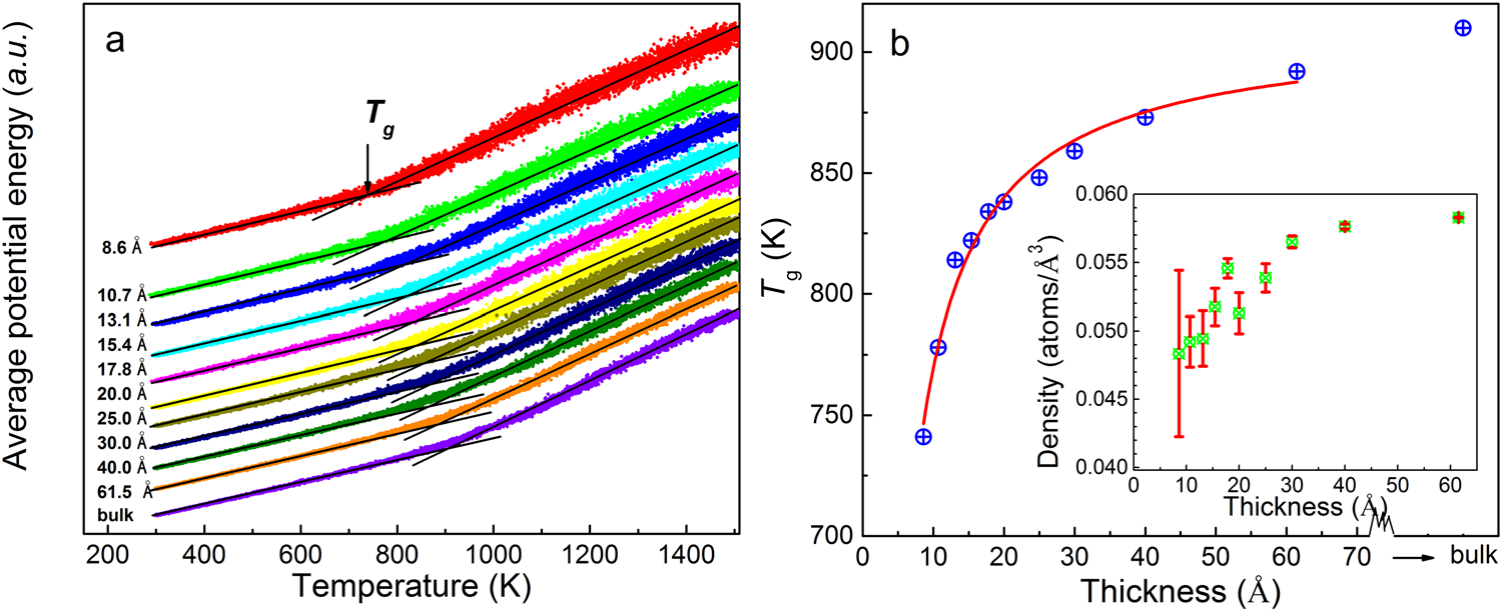
(**a**) Determination of glass transition temperature *T*_g_ via an intersection of low- and high-temperature dependences of the potential energy in Cu_64_Zr_36_ SSAFs with different thicknesses and bulk Cu_64_Zr_36_ MG. (**b**) The film thickness dependent *T*_g_ of Cu_64_Zr_36_ SSAFs with different thicknesses together with the fitting curve, which is explained in text. And the inset picture is atomic number density as a function of film thickness for Cu_64_Zr_36_ SSAFs.

To further explore the size effects on atomic structure and glass transition temperature of MGs, we then extend our studies to free standing two-dimensional thin amorphous films. Figure 7 shows the thickness-dependence of pair distribution function *g*(*r*) curves for Cu_64_Zr_36_ SSAFs at 1500 K and 300 K, respectively. Compared to the liquid state at 1500 K, the first peaks at 300 K for all the thin films tend to be sharper, suggesting much less vibration for atoms at lower temperature. The broadened peaks for films demonstrate that all studied thin films are in amorphous state at 300 K. Figure 8a shows the average atomic internal energy of Cu_64_Zr_36_ SSAFs with various thicknesses as a function of temperature during cooling. *T*_*g*_ for Cu_64_Zr_36_ SSAFs with various thicknesses was estimated as the intersection point of the two solid lines, as in Fig. 8a. Figure 8b depicts the glass transition temperature as a function of film thickness for Cu_64_Zr_36_ SSAFs together with the bulk MG sample. It is found that the *T*_*g*_ decreases substantially as the film thickness decreases compared to the bulk MG sample. As plotted in a red solid curve, we fitted the data to a function of, where *T*_*g*_ *(d)* is glass transition temperatures of *d*-thickness SSAFs, *T*_*g*_ *(∞)* and C are constant fitting parameters. Then, we obtained *T*_*g*_ *(∞)* as 909 K which is approximately equal to the *T*_*g*_ of Cu_64_Zr_36_ BMG and the fitted value of C was 0.155. The densities of Cu_64_Zr_36_ SSAFs are also plotted in inset of Fig. 8b as a function of film thickness, which decreases with the reduction of film thickness. The reduction of density strongly suggests larger motion of atoms in thinner SSAFs, which may contribute to the reduced *T*_*g*_. To understand this thickness dependent *T*_*g*_ of Cu_64_Zr_36_ SSAFs, we further analyze the contribution of the surface layer in SSAFs.

### Composition and MSD variations in core and surface layers for Cu_64_Zr_36_ SSAFs

**Figure 9:**
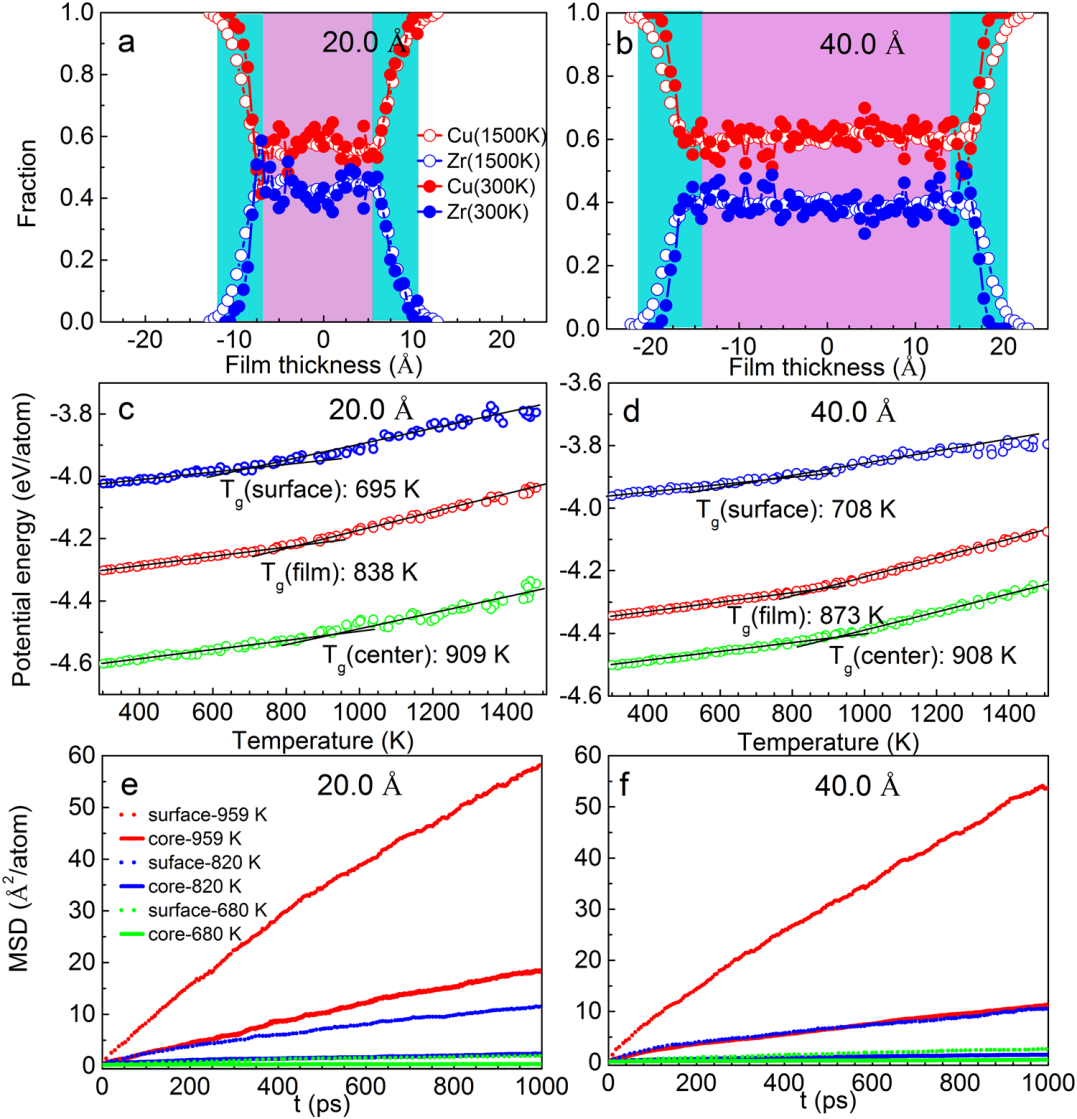
Composition variations along the film thickness for Cu_64_Zr_36_ SSAFs with thicknesses of (**a**) 20.0 Å and (**b**) 40.0 Å. Determination of glass transition temperature *T*_g_ (total, core and surface components) of (**c**) 20.0 Å and (**d**) 40.0 Å thick Cu_64_Zr_36_ SSAFs. Mean atomic square displacements at two regions (core and surface) of (**e**) 20.0 Å and (**f**) 40.0 Å thick Cu_64_Zr_36_ SSAFs at 680, 820 and 959 K.

In all studied SSAFs, it is found that the films can be composed of two regions, i.e., the core region and surface layer region with a thickness of about 0.7 nm, which is estimated from the thickness dependent composition profiles. We also revealed that surface region has an impact on the thermal properties of SSAFs, such as glasses transition temperature and dynamic motion. Figure 9a and b demonstrate the composition variations along the film thickness for two selected 20 Å and 40 Å thick Cu_64_Zr_36_ SSAFs. Similar feature is also detected in Cu_64_Zr_36_ SSAFs with other thicknesses. In the blue region as the surface layer, the fraction of Cu atoms is found to be much more than the assigned alloy of 64 at.%, indicating that Cu atoms segregate to the surface which is consistent with initial studies^55^ and results reported for our above-studied Cu_64_Zr_36_ SSAPs. In the pink region as the core layer, its composition fluctuation is much smaller and close to the average value of 64 at.%. Moreover, it has been reported that the liquid-glass transition temperature increases as the Cu concentrated in the region of 0.348 < *x*_Cu_ < 0.649 and then decreases^56^. The surface layers for all studied Cu_64_Zr_36_ SSAFs have a similar value of *T*_*g*_ = 812 ± 2 K, which is lower than 908 ± 2 K for the central layer, which is also similar value in all studied Cu_64_Zr_36_ SSAFs, as illustrated in Fig. 9c and d. With decreasing of film thickness, the surface layers make more contribution on *T*_*g*_ of the total films due to the larger surface-to-volume ratio for thinner films. Consequently, the thinner the film thickness, the lower *T*_*g*_ of the SSAF. Since the surface layer plays an important role in affecting the glass transition temperature of SSAFs, we carried out a further MD calculation to investigate the dynamic properties of different regions. The samples are quenched with a cooling rate of 1 × 10^12^ K/s from 1500 K to fixed temperature, which the atoms relaxed sufficiently: T = 680 K, 820 K and 959 K. The mean square displacement (MSD) profiles as a function of time t for central and surface regions are calculated according to the equation:

3 $$MSD=\,\frac{1}{N}\langle \sum _{i}^{N}{[{r}_{i}(t)-{r}_{i}({t}_{0})]}^{2}\rangle $$

where *r*_*i*_ (t) refers to the position of the atom *i* at the relaxed time t, *r*_*i*_ (t0) is the position of the corresponding atom at original time t_0_ and N is the total atom number of the corresponding region. In Fig. 9e and f show, it is remarkable that the values of MSD and the slopes of MSD *vs* time in the surface layer are larger than those in the central layer at three selected temperatures, suggesting that the mobility of atoms in the surface layer are faster than that in the central layer. The higher atomic mobility in surface layer should be linked to its atomic structure and composition.

In conclusion, atomic structures in Cu_64_Zr_36_ SSAPs with a range of 50–5000 atoms and Cu_64_Zr_36_ SSAFs with a thickness range of 8.6–61.5 Å have been systematically investigated by molecular dynamics simulations. We uncovered that the sample size (particle size and film thickness) indeed influences the atomic structure of Cu_64_Zr_36_ SSAPs and SSAFs, in which two different parts, i.e., core and shell (or surface) components, can be characterized. For Cu_64_Zr_36_ SSAPs, in the shell component, the average coordination number of about 8.5, the average bond length of 2.7 Å and higher degree of ordering, i.e., larger *Q*_*4*_ and *Q*_*6*_, were detected while the average coordination number of about 12.5, the average bond length of 2.9 Å and lower degree of ordering, i.e., lower *Q*_*4*_ and *Q*_*6*_, were observed in the core component. The atomic packing in the core component with high fraction of the fivefold symmetry is denser than that in the shell component with high fraction of 1321 HA index symmetry. These atomic structure differences in Cu_64_Zr_36_ SSAPs with various sizes affect their glass transition temperatures, which can be elucidated by relative contributions of both core and shell components. The glass transition temperature for the shell component is found to be 577 K, much lower than 910 K for the core component. For Cu_64_Zr_36_ SSAFs, we revealed that *T*_g_ decreases as the metallic glass films get thinner. It has been manifested unambiguously that free surface is the dominant cause for *T*_g_ reductions. Cu atoms segregate to the surface causing a far higher concentration while the central part almost remains the same composition as films. It is observed that atoms in surface layer have larger mobility than central part and they require lower temperatures to reach a glass state during cooling, i.e., lower *T*_g_ for the surface layer. All in all, due to the different behaviors of shell (or surface) component and core composition in Cu_64_Zr_36_ SSAPs and SSAFs, the size effects on atomic structure and *T*_*g*_ are clearly demonstrated. The lower the particle size (or film thickness), the lower *T*_*g*_. The finding reported here promotes our understanding of the size effect on atomic structure in amorphous systems, which will trigger more studies on the unsolved puzzle of atomic structure in disordered materials in general.

## Methods

### Molecular dynamics simulations

Molecular dynamics simulations of Cu_64_Zr_36_ SSAPs and SSAFs were performed based on the embedded atom method (EAM) potential^38^ in the canonical Ensemble (NVT) using the large-scale atomic molecular massively parallel simulator (LAMMPS) code^57^. For SSAPs, the spherical particles having various atom numbers of 50, 100, 200, 400, 700, 1000, 4000, 5000 and their corresponding radii of about 5.5 Å, 7.4 Å, 9.5 Å, 12.1 Å, 14.8 Å, 17.2 Å, 27.0 Å, 29.5 Å, respectively, are constructed. These spherical particles are then placed at the center of simulation box with sizes of 62 Å for 50–1000 atoms, and 80 Å for 4000 and 5000 atoms to achieve a sufficient vacuum layer and negligible interaction between SSAPs when the simulation box is under the periodic boundary condition. These nanoparticles were firstly melted and equilibrated at 1500 K for 1 ns to eliminate the influence of initial structure and cooled to 300 K with a cooling rate of 1 × 10^12^ K/s. They were equilibrated at 300 K for 1 ns and exhibited amorphous structure, i.e., without long-range period ordering. We further analyzed atomic structures of all studied SSAPs with various sizes, in which two components, i.e., core and shell, were divided. In order to obtain reliable structural information, all structure analyses with 2000 configurations were carried to get average values. For comparison, atomic structure of a bulk Cu_64_Zr_36_ MG was also studied.

For SSAFs, the model system of Cu_64_Zr_36_ MG with a 62 Å × 62 Å × 62 Å box and ~13500 atoms was first generated. The cubic cell was melted at 2000 K which is far above the melting point of the amorphous system and kept at zero pressure for 2 ns to ensure homogeneity, during which periodic boundary conditions were applied to all three dimensions. The simulation cell was then quenched from 2000 K to 1500 K at 1 × 10^12^ K/s and relaxed for 1 ns. Thin film melts with different thicknesses are obtained by cutting the bulk box to the given thicknesses from 8.6 Å to 61.5 Å and set two vacuum layers along the thick direction to reach free boundary condition. After relaxing the thin films for 1 ns at 1500 K, all the systems were quenched down to 300 K with a cooling rate of 1 × 10^12^ K/s and relaxed for 1 ns.

### Pair distribution functions in small size systems

As a useful tool for local structure determination, the pair distribution functions (PDF) can be applied to analysis the structure of nanoparticles and thin films. However, it is hard to determine their structure by ordinary techniques used for bulk materials because the periodicity of them is limited. PDF programs were developed to fit the SSAPs and SSAFs. Below details are described only for SSAPs. PDF (denoted by $g(r)$) describes how density varies as a function of distance from a reference atom. Taking a system composed of one kind of atoms, PDF is defined as the probability to find an atom in a shell ${dr}$ at the distance *r* of another atom chosen as a reference point. By dividing the physical model volume into shells ${dr}$ it is possible to compute the number of atoms ${dn}(r)$ at a distance between $r$ and $r+{dr}$ from a given atom:

4 $$dn(r)=\frac{N}{{V}_{0}}g(r)dV(r)$$

where $N$ represents the total number of atoms, ${V}_{0}$ the model volume. In this notation the volume of the shell of thickness ${dr}$ is approximated by:

5 $$dV(r)=\mathop{\mathrm{lim}}\limits_{dr\to 0}(\frac{4}{3}\pi {(r+dr)}^{3}-\frac{4}{3}\pi {r}^{3})=4\pi {r}^{2}dr=Sdr$$

where $S$ is the surface area of the shell. It should be noted that this equation is only applicable to extended bulk materials. For finite-sized materials, such as nanoparticles, surface area should be modified according to their size and shape. Finally, PDF could be obtained via:

6 $$g(r)=\frac{dn(r)}{dV(r)\ast \frac{N}{{V}_{0}}}=\frac{1}{S}\frac{{V}_{0}}{N}\frac{dn(r)}{dr}$$

**Figure 10:**
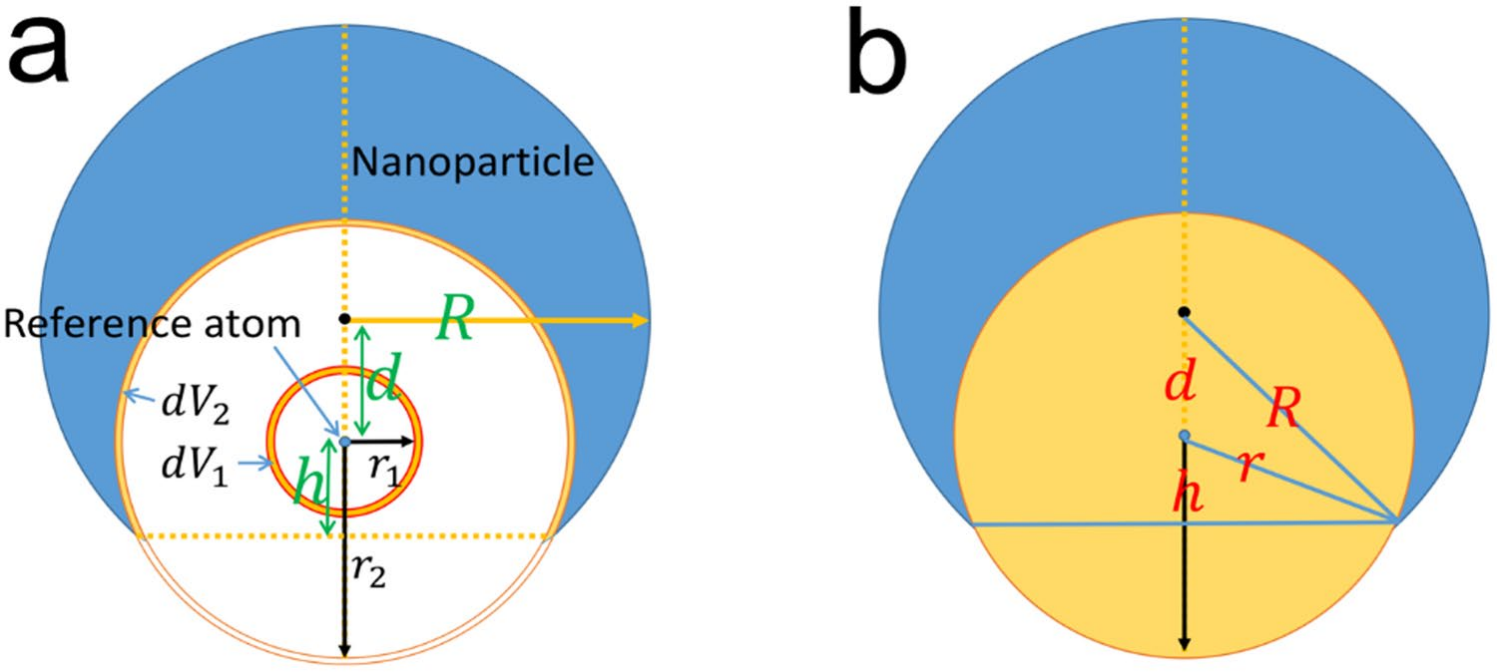
Sectioned diagram of a spherical particle. (**a**) Shell with radius $r$ locates inside the nanoparticle. (**b**) Surface area of overlapped shell.

Though PDF technique has been well developed for extended bulk materials, the corrections for the size and shape effect must be considered in finite-sized nanoparticles. In this section, we consider a spherical nanoparticle with radius $R$ and number of atoms $N$ shown in Fig. 10. The volume of this nanoparticle is obtained as

7 $${V}_{0}=\frac{4}{3}\pi {R}^{3}$$

${dn}(r)$, number of atoms in the shell of $r\to r+{dr}$, is counted directly as in bulk materials. However, the volume of shells which overlaps with nanoparticle ${dV}(r)={S}^{^{\prime} }{dr}$ is different from the bulk situation^58, 59^.

8 $$S^{\prime} (r)=\{\begin{array}{c}4\pi {r}^{2}\quad \quad \quad \quad \quad \quad \,(r+d\le R)\\ 2\pi r(h+d),\,h=\frac{{R}^{2}-{r}^{2}-{d}^{2}}{2d}\,\quad (r+d > R)\end{array}$$

where $d$ is the distance between center of nanoparticle and the reference atom. When shell with radius $r$ locates inside the nanoparticle ($r+d\le R$), e.g., ${r}_{1}$ as shown in Fig. 10a, the surface area is $4\pi {r}^{2}$. However, when part of the shell locates outside of the nanoparticle ($r+d > R$), only the overlap part of shell should be counted (upper part of shell with radius ${r}_{2}$ in Fig. 10a). The surface area of overlapped shell could be calculated with geometry relations depicted in Fig. 10b. Finally, $g(r)$ of spherical nanoparticle is obtained via:

9 $$g(r)=\frac{1}{S^{\prime} (r)}\frac{{V}_{0}}{N}\frac{dn(r)}{dr}$$
